# Evaluation of Two Combined Analytical Approaches to Assess the Impact of Rare Variants on Severe COVID-19

**DOI:** 10.3390/ijms27209152

**Published:** 2026-10-15

**Authors:** Francesca Rosamilia, Giada Recchi, Davide Cangelosi, Sergio Decherchi, Francesca Lantieri, Stefania Croci, Carlo Salvarani, Andrea Cavalli, Marta Rusmini, Isabella Ceccherini, Paolo Uva

**Affiliations:** 1 Clinical Bioinformatics Unit, IRCCS Istituto Giannina Gaslini, 16147 Genoa, Italy; francescarosamilia@gaslini.org (F.R.); paolouva@gaslini.org (P.U.); 2 Rheumatology and Autoinflammatory Diseases Unit, IRCCS Istituto Giannina Gaslini, 16147 Genoa, Italy; giadarecchi@gaslini.org; 3 Artificial Intelligence Unit, IRCCS Istituto Giannina Gaslini, 16147 Genoa, Italy; davide.cangelosi@gaslini.org; 4 Data Science and Computation Facility, Fondazione Istituto Italiano di Tecnologia, 16163 Genoa, Italy; sergio.decherchi@iit.it; 5 Biostatistics Unit, Department of Health Sciences (DISSAL), University of Genoa, 16132 Genoa, Italy; f.lantieri@unige.it; 6 Autoimmunity, Allergology and Innovative Biotechnology Unit, AUSL-IRCCS of Reggio Emilia, 42123 Reggio Emilia, Italy; stefania.croci@ausl.re.it (S.C.); carlo.salvarani@ausl.re.it (C.S.); 7 Computational and Chemical Biology, Fondazione Istituto Italiano di Tecnologia, 16163 Genoa, Italy; andrea.cavalli@epfl.ch; 8 CECAM-EPFL, 1015 Lausanne, Switzerland; 9 UOSD Research Laboratories Aggregation Area, IRCCS Istituto Giannina Gaslini, 16147 Genoa, Italy; isabellaceccherini@gaslini.org

**Keywords:** COVID-19, rare variants, machine learning

## Abstract

Severe COVID-19 displays substantial inter-individual variability that cannot be fully explained by common genetic variants, suggesting a contribution of rare functional variation. We performed whole-genome sequencing in 191 Italian patients (96 moderate, 95 severe) to investigate rare coding variants (minor allele frequency <1% and <5%) via complementary network-based and machine learning approaches. Classical single-variant and burden tests did not identify significant associations after multiple testing correction. However, Network-based Heterogeneity Clustering (NHC) revealed that multiple gene clusters were enriched in severe cases, particularly those involving RNA polymerase II–mediated transcription, RNA metabolism, and vesicle-mediated transport pathways. Gene-level rare variant analysis via the variable threshold method identified five genes significantly associated with severe disease (FDR < 0.05), namely, *EIF4G3*, *GTF2F2*, *LY96*, *COX6A1*, and *STK11*, which are involved in transcriptional and translational regulation, innate immune signaling, and mitochondrial function. Unsupervised biclustering significantly stratified patients by severity, and a decision tree model highlighted the burden of *DOCK10* variants as a key discriminator between phenotypes. Convergence between the network-based and machine learning results further supported the robustness of the identified signals. Overall, our integrative framework underscores the contribution of rare variants to COVID-19 severity and highlights the coordinated perturbation of interconnected molecular pathways as a determinant of clinical outcome.

## 1. Introduction

The coronavirus disease 2019 (COVID-19) pandemic, caused by the SARS-CoV-2 virus, has profoundly impacted the lives of millions of people worldwide. Owing to its wide-ranging effects on health and society, COVID-19 has become the focus of intense scientific research. Identifying the biological and genetic factors influencing disease severity has emerged as a critical objective in understanding individual susceptibility and improving clinical outcomes.

Several studies have revealed a correlation between disease severity and known risk factors, such as male sex [1]; advanced age [2,3]; and comorbidities, including cancer, diabetes, autoimmune disorders or immunodeficiency, coronary artery disease, obesity, and hypertension [4,5,6,7]. Additionally, the presence of preexisting infections or autoantibodies has been associated with worse outcomes [8,9]. However, severe disease has also occurred in individuals without these known risk factors, suggesting a role for genetic predisposition [10,11].

COVID-19 is now considered a complex disease with both genetic and environmental contributors [12,13]. Genome-wide association studies (GWASs), especially those coordinated through large international consortia such as the COVID-19 Host Genetics Initiative (HGI) [14] and the COVID-19 Human Genetic Effort [15,16], have successfully identified common genetic variants associated with disease susceptibility and severity. One of the most significant findings is the 3p21.31 locus, which is known to contain a Neanderthal-derived haplotype associated with severe infection [17,18]. Other loci include genes involved in the immune response (e.g., *IFNAR1*, *TYK2*, and *OAS1/2/3*) and lung function (e.g., *MUC5B* and *DPP9*) [19,20,21,22,23]. Moreover, we identified C-reactive protein (CRP) as a critical biomarker and genetic determinant of COVID-19 severity. In particular, Mendelian randomization further supported a causal role of common variants in genes associated with elevated CRP levels and disease outcomes [24].

In addition to common variants, rare genetic variants—those occurring in less than 1–5% of the population—have also been shown to influence COVID-19 severity. These variants can have a profound impact on protein function and host immunity [25,26]. For example, rare loss-of-function mutations in *TLR7*, *IRF7*, and *IRF9* disrupt type I interferon signaling and are enriched in unvaccinated individuals with life-threatening COVID-19 pneumonia. Studies have estimated that such variants may explain up to 5% of critical cases, highlighting the role of inborn errors in immunity [27,28].

Despite these findings, the identification and statistical evaluation of rare variants remain challenging. While univariate burden tests can assess the effects of individual genes, multivariate methods can capture interactions and cumulative effects. Both approaches offer complementary insights into the genetic architecture of complex traits [29,30,31].

In this study, we investigated the contribution of rare genetic variants to COVID-19 severity in a small yet clinically and geographically homogeneous cohort of Italian patients. Two complementary analytical strategies were employed: first, pathway enrichment analysis followed by a gene-level association test to identify potential correlations; second, a machine learning approach integrating unsupervised biclustering with a decision tree classifier to uncover patterns of genetic contribution among clinical subgroups was used. Together, these methods provide a deeper understanding of the role of rare genetic variants in the severity of COVID-19.

## 2. Results

**Figure 1:**
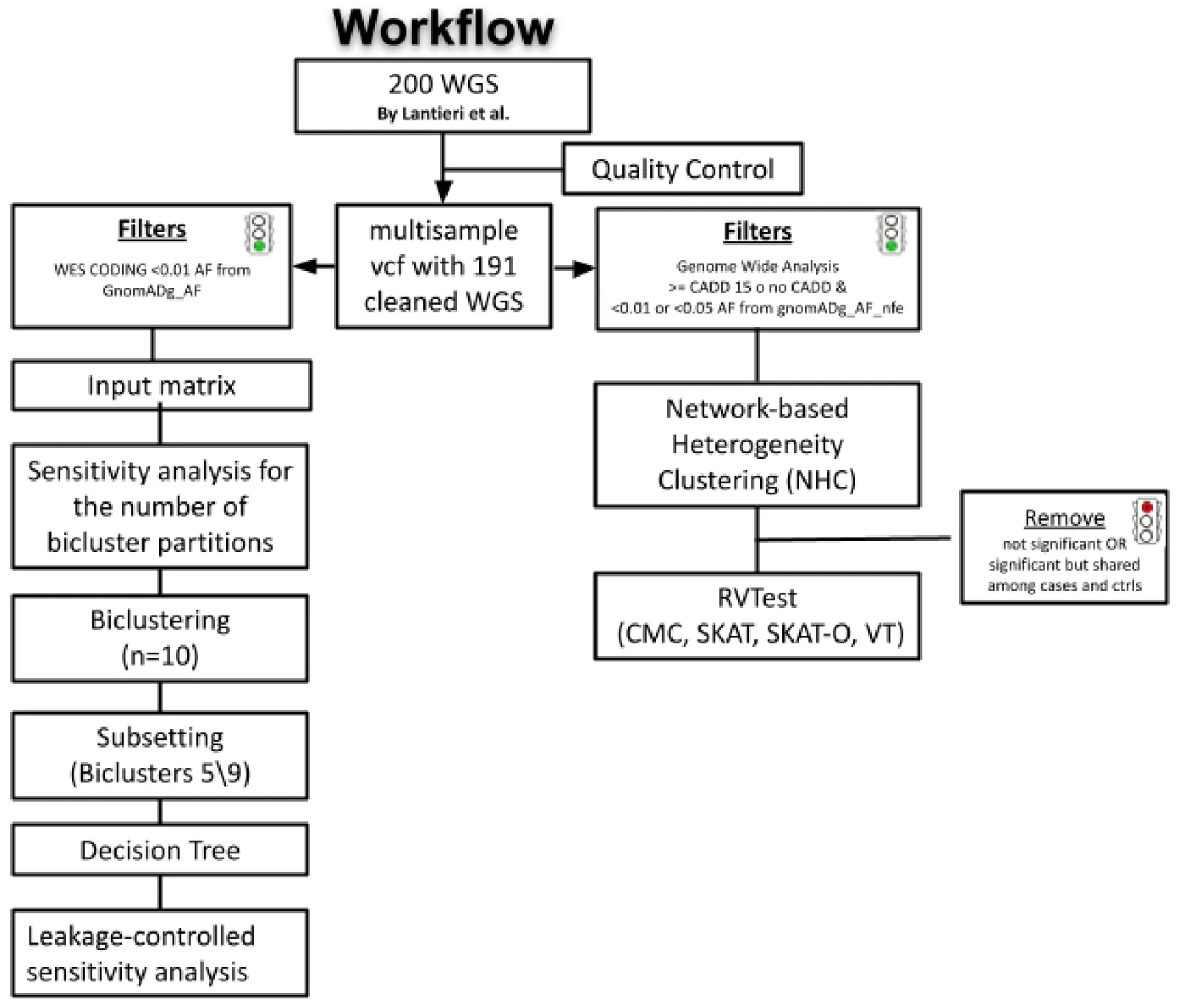
Workflow outlining the sequential steps from variant processing and statistical analysis to machine learning–driven patient clustering and classification. The dataset under study here was generated as reported by Lantieri et al. (2026) and already used for the analysis of common variants (freq. > 5%) [24].

An overview of the study workflow is provided in Figure 1, which shows the sequential steps from variant processing and statistical analyses to machine learning–based patient clustering and classification.

### 2.1. WGS Analysis Results

The mean coverage of each WGS was >30x, with >85% of the genome covered at ≥20x depth.

After data cleaning, 9 samples were removed because they failed one of the following filters: non-European ancestry, genotype missingness higher than 3% or abnormal heterozygosity (above the mean ±3 SD). The following analyses were thus performed on the remaining 191 samples.

A total of 645,679 rare variants (MAF < 0.01) located in the coding region ±100 bp were identified. The most common variant types were intronic (35.15%), 3′UTR (16.87%), and missense (9.08%) variants, as reported, together with the full breakdown of variant types, in Supplementary Table S1.

A targeted analysis was performed to compare the severe and moderate patient groups. Variants were filtered to exclude small insertions and deletions (Indels) and those variants at single nucleotides (SNVs) with low impact annotations, as well as variants classified as benign/likely benign according to ClinVar or with a CADD score < 15. No variant was observed in more than 2 individuals (2%) in one group, and it was completely absent in the other group.

Because the rare-variant analysis restricted to the predefined gene panel yielded no informative signals, the single-variant approach was discontinued, and the analysis was broadened to include all rare variants across the coding region aggregated at the gene level.

Consistent with the initial findings, no individual rare variant was found to be exclusive to either the severe or moderate group. Therefore, we applied a burden test via Fisher’s exact test in R, v.4.3.1 [32]. After evaluating the per-gene enrichment of rare variants between the two groups and correcting for multiple testing, no gene reached statistical significance.

### 2.2. Biclustering Analysis for Patient Stratification

To explore hidden structures in the data and stratify patients according to disease severity, a Python-based workflow was developed and employed to analyze patient data (2,353,113 features and 191 patients). Biclustering analysis was unsupervised and thereby did not include disease severity.

Stability analysis (Supplementary Table S2) showed that the five-cluster solution had the highest conservative joint ARI (Adjusted Rand Index). However, this solution was nearly degenerate, with 180 of 191 participants assigned to one cluster and a median patient-cluster size of one. The ten-cluster solution provided the most stable variant partition, with mean ARIs of 0.760 across initializations and 0.706 following 80% participant subsampling. Corresponding patient-partition ARIs were lower, at 0.162 and 0.100, respectively. Increasing the resolution to 15 clusters did not improve stability, yielding subsampling ARIs of 0.073 for patients and 0.694 for variants. We therefore retained K = 10 as an intermediate resolution rather than as a uniquely optimal number of clusters.

**Figure 2:**
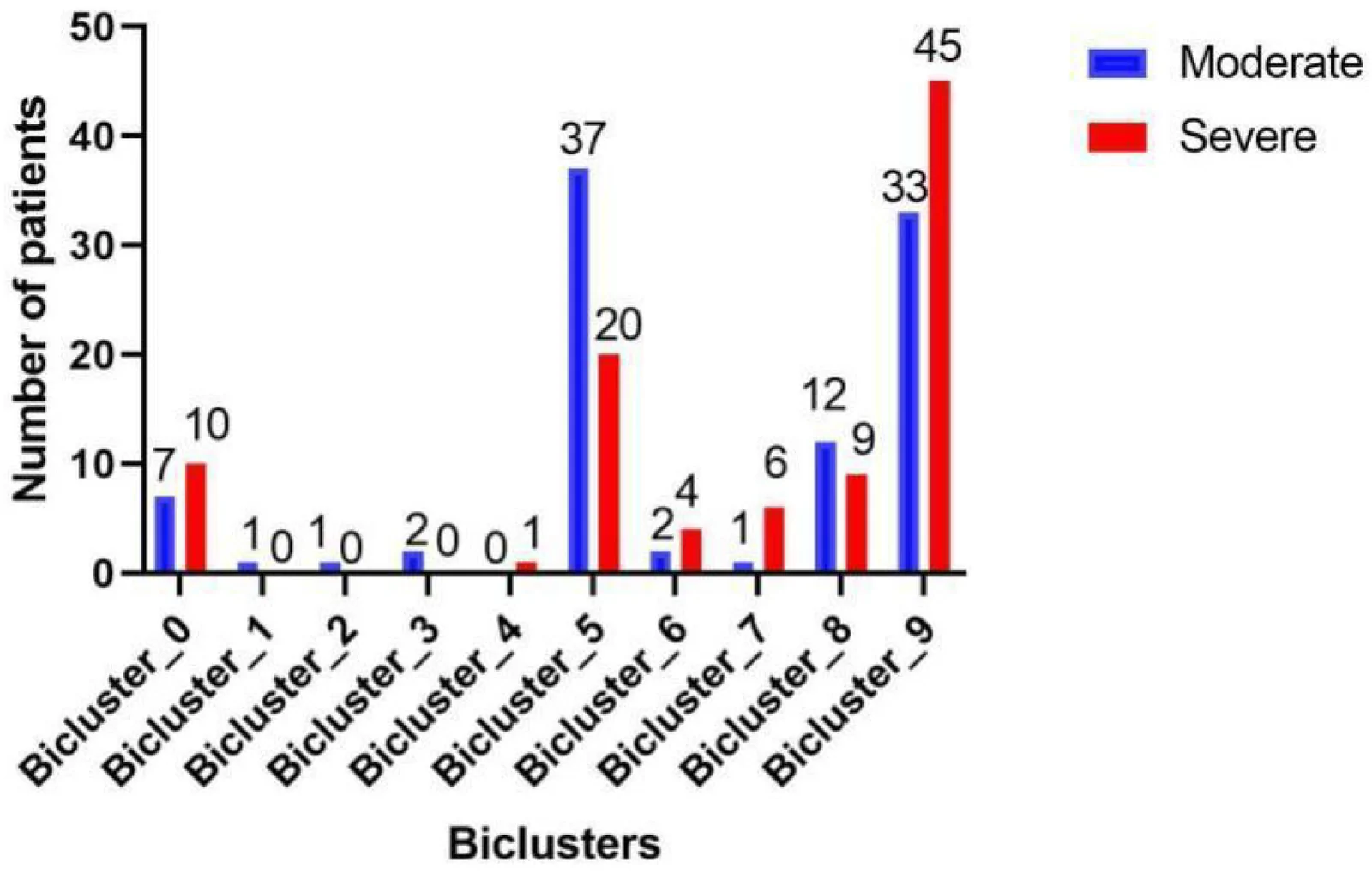
Bar plots of the distribution of severe/moderate phenotypes across biclusters. The bar plot shows the number of patients with severe (red) and moderate (blue) phenotypes on the y-axis and the bicluster number on the x-axis. The number of patients is reported above each bar. Phenotypes are displayed in the top-right part of the plot.

Spectral biclustering identified the prespecified checkerboard structure comprising 10 patient row clusters and 10 variant column clusters (Figure 2). Patient row-cluster membership was associated with disease severity in the unadjusted contingency-table analysis (Fisher’s exact test with Monte Carlo approximation, *p* = 0.020).

Of the 191 participants, 183 had complete information on disease severity, row-cluster membership, sex, age, and comorbidity and were included in the multivariable analysis. Eight participants were excluded because comorbidity status was missing. After adjustment for sex, age, and comorbidity, inclusion of row-cluster membership significantly improved model fit compared with the model containing the clinical covariates alone (likelihood-ratio χ^2^ [9] = 18.08, *p* = 0.034). These results indicate that the overall association between patient clustering and disease severity was not fully explained by the measured differences in sex, age, or comorbidity.

Among the biclusters identified, two (5 and 9) included the majority of patients, that is, 135 of the 191 (70.6%). Bicluster 9 (78 patients, 40.8%) was enriched for severe cases (45 of 78; 57.7%), whereas Bicluster 5 (57 patients, 29.8%) was predominantly composed of moderate cases (37 of 57; 64.9%). Together, these two biclusters encompassed 3044 of the 2,353,113 features (0.13%) that were used to train a decision tree classifier. The optimized model (fivefold cross-validation; criterion = Gini, max_depth = 4, min_samples_leaf = 5) generated a tree whose root split was driven by the number of variants lacking a CADD score in the *DOCK10* gene. In particular, 65 severe and 70 moderate cases are explainable by the number of variants in *DOCK10*: fewer than 14 variants characterize moderate cases, whereas the opposite characterizes severe cases, highlighting *DOCK10* as the primary discriminator between phenotypic groups.

### 2.3. Sensitivity Analysis of the Generalizability of the Bicluster-Derived Features

In nested cross-validation, predictive discrimination was close to chance, with an out-of-fold ROC-AUC of 0.504 (95% CI, 0.420–0.587), balanced accuracy of 0.508 (95% CI, 0.437–0.575), and MCC of 0.017 (95% CI, −0.129 to 0.151). These findings indicate that the variant rankings obtained from the complete cohort should be regarded as characteristics of the observed biclusters rather than as a validated predictive signature.

### 2.4. NHC Application in Severe vs. Moderate COVID-19 Groups

For the joint multisample VCF of 191 individuals, NHC was applied via high-quality and high-impact rare variants (see Methods).

Two thresholds were tested:

- Very rare (gnomADg_AF_nfe < 1%): 32 gene clusters significantly enriched in severe cases, each associated with unique pathways (*p* ≤ 1 × 10^−5^).
- Rare (gnomADg_AF_nfe < 5%): identified 29 gene clusters, again pathway-enriched but only significant in severe vs. moderate (not vice versa).

Notably, these latter gene clusters were significant in the severe versus moderate case–control group but not in the reverse group (Supplementary Table S3). This is because NHC does not intrinsically assign directionality to enrichment: large clusters or pathways may contain different sets of mutated genes in each group, leading to apparent enrichment in both directions. Therefore, both contrasts were tested, and only clusters significant in the severe vs. moderate comparison but not in the reverse comparison were retained. Among the prioritized genes, “RNA polymerase II transcribes snRNA genes” stood out (*p* = 5.468 × 10^−23^), along with “reactome COPII-mediated vesicle transport” (*p* = 1.043 × 10^−16^) and “reactome, membrane trafficking” (*p* = 1.782 × 10^−12^). These *p* values reflect enrichment within a gene set selected from the same cohort and should be interpreted as post-selection exploratory results rather than as formal tests of association with severity.

All genes identified in the enriched pathways were investigated for their association with COVID-19 via a PubMed e-utilities analysis. Among them, ACE (located in cluster 21 of Supplementary Table S3) was the most frequently cited gene, with 1894 publications. Notably, the “Reactome acetylcholine neurotransmitter release cycle” pathway (cluster 16) was strongly enriched (*p* = 4.41 × 10^−13^) and has previously been reported to be associated with COVID-19 [33]. Furthermore, of the 702 prioritised genes with *p* < 0.05 (Supplementary Table S3), 86 were previously associated with various traits in the GWAS catalog [34], and 31 of these genes were linked to multiple traits (e.g., asthma, HDL levels, and type 2 diabetes). This overlap provides preliminary, hypothesis-generating context for the prioritised genes, but does not by itself validate them.

### 2.5. NHC Gene Association

**Figure 3:**
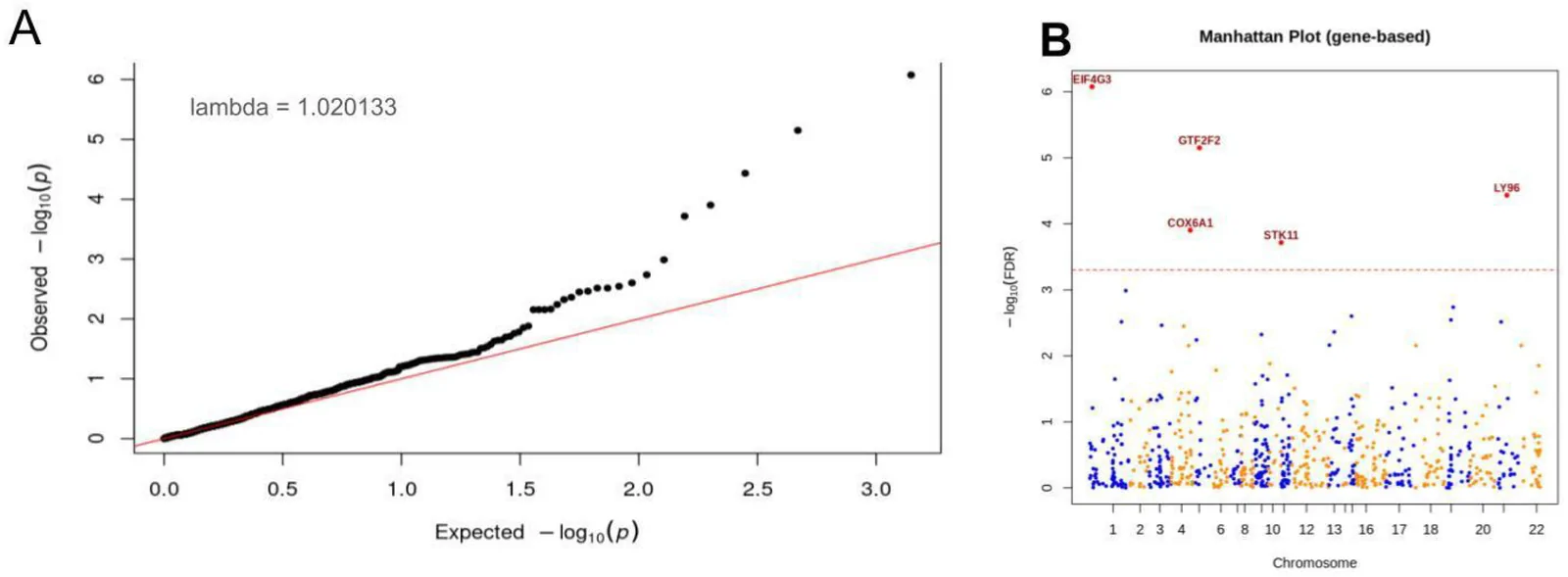
(**A**) Representation of the genomic inflation factor (λ) of the whole *p* value resulting from the variable threshold method analysis. (**B**) Manhattan plot representing the *p* value (−log10(FDR)) of each gene, analyzed via the variable threshold method. The dotted line represents the threshold; the statistically significant genes are *EIF4G3*, *GTF2F2*, *LY96*, *COX6A1* and *STK11*.

Rare variant association testing (CMC, SKAT, SKAT-O, and variable threshold) was conducted on the 702 NHC-prioritized genes. Only the variable threshold method yielded statistically significant results. The genomic inflation factor (λ) of the whole *p* value resulting from this method confirmed that the data were not inflated (λ = 1.02) (Figure 3A).

Statistical analysis (Supplementary Table S4) via the variable threshold method of Price et al. [35], which groups rare variants by selecting the allele–frequency threshold that maximizes the z score difference between cases and controls, revealed five statistically significant genes, namely, *EIF4G3*, *GTF2F2*, *LY96*, *COX6A1* and *STK11*, as reported in Figure 3B and in Table 1.

For each gene, the table reports the total number of analyzed variants (NumVar) and the number of polymorphic variants (NumPolyVar). OptFreq indicates the optimal allele frequency threshold selected by the NHC procedure. Zmax represents the maximum standardized test statistic obtained across frequency thresholds, and Stat is the corresponding observed NHC test statistic. NumGreater and NumEqual denote the number of permutation replicates with test statistics greater than or equal to the observed statistic, respectively. The Perm*p* value is the empirical permutation-based *p* value calculated from these replicates. Fdr.*p* corresponds to the false discovery rate (FDR)-adjusted *p* value accounting for multiple testing across genes. Only statistically significant genes (fdr.*p* < 0.05) are reported.

Human gene expression was assessed in silico via publicly available datasets for the above statistically significant genes. In particular, we interrogated the Human Protein Atlas [36] and the GTEx portal [37] to determine whether these genes are ubiquitously expressed and to evaluate their potential as candidate genes.

*STK11*, encoding serine/threonine kinase 11, is ubiquitously expressed across tissues but shows relative cellular specificity in immune cells, particularly neutrophils. *COX6A1* encodes a subunit of cytochrome c oxidase (complex IV), the terminal enzyme of the mitochondrial electron transport chain responsible for reducing oxygen to water and driving oxidative phosphorylation; it is ubiquitously expressed at relatively high levels in brain tissue and localizes to mitochondria. *LY96* encodes lymphocyte antigen 96, a coreceptor of Toll-like receptor 4 that mediates responsiveness to lipopolysaccharide (LPS); it is expressed mainly in lymphoid tissues and contributes to immune response regulation. *GTF2F2* encodes a subunit of the general transcription factor IIF involved in RNA polymerase II–mediated transcription and displays low tissue specificity. Finally, the most statistically significant gene, *EIF4G3*, also has low tissue specificity and encodes a component of the eIF4F complex, which regulates mRNA cap recognition and recruitment to the ribosome during translation initiation.

The genes whose features were used by the machine learning algorithm to identify biclusters intersected with the statistically significant genes from the NHC analysis. There were 30 genes in common, which are underlined in Supplementary Table S4.

Among these genes, the *EIF4G3* gene, the first to be significantly different among the NHC genes, and its variants contributed to the determination of bicluster 5.

## 3. Discussion

Understanding the contribution of rare and ultrarare genetic variants to COVID-19 severity represents a substantial analytical challenge due to data sparsity, high dimensionality, and cohort heterogeneity. Traditional rare variant association methods have been largely developed for Mendelian disorders and are often insufficient to capture the complex, nonlinear architecture underlying multifactorial phenotypes such as COVID-19 severity [38]. Since rare variants may account for part of the missing heritability not explained by common GWAS loci [39], developing computational strategies capable of extracting robust signals from limited datasets is essential.

To address these challenges, we implemented an integrative analytical framework that combines biologically informed network aggregation with interpretable machine learning. This dual strategy was designed to increase statistical power, improve biological interpretability, and capture both coherent pathway-level signals and nonlinear combinatorial genetic effects.

The network-based heterogeneity clustering (NHC) approach integrates protein–protein interaction networks with rare variant distributions to identify functionally coherent gene clusters. This network-aware strategy may be particularly useful in small or genetically heterogeneous cohorts, where single-gene analyses may lack power [40]. In our study, NHC prioritised pathways involved in RNA polymerase II–mediated snRNA transcription and COPII-mediated vesicle transport among severe cases (Supplementary Table S3) [41]. These processes are known to be perturbed during viral infections and are central to transcriptional regulation and intracellular trafficking [42]. This suggests that contextualizing rare variants within biological networks may help generate hypotheses for higher-order functional modules potentially relevant to disease severity.

Gene-based burden testing via the variable threshold was additionally performed on the genes selected by NHC analysis and highlighted genes with a higher burden in severe cases, including *EIF4G3* and *GTF2F2* (involved in transcription and translation regulation) [43,44,45], *LY96* (a co-receptor in innate immune signaling) [46,47], *COX6A1* (a mitochondrial respiratory chain component) [48,49] and *STK11* (a regulator of cellular metabolism and stress responses) [50,51]. Because these genes were prioritised and tested in the same cohort, all gene and pathway findings should be regarded as post-selection exploratory hypotheses.

Although these genes span diverse molecular functions, they converge on interconnected biological processes—transcriptional control, immune sensing, mitochondrial metabolism, and cellular stress adaptation—suggesting that coordinated perturbation of host regulatory networks, rather than isolated gene defects, contributes to severe COVID-19 phenotypes.

To further capture complex genotype–phenotype relationships beyond additive burden effects, we applied unsupervised biclustering to identify subgroups of patients characterized by co-occurring rare variants, followed by an interpretable decision tree classifier [52,53]. This approach addresses a key issue in health informatics: the need to balance modeling with interpretability. Biclustering identified subgroups of patients characterized by co-occurring rare variants across specific gene sets, and the subsequent decision tree organized these features into an interpretable hierarchical model. Importantly, the overall association between patient row-cluster membership and disease severity remained statistically significant after adjustment for sex, age, and comorbidity. Nevertheless, this finding should be considered exploratory because of the small size of some patient clusters and the possibility of residual confounding.

Notably, 30 genes overlapped between those defining biclusters and those identified through NHC analysis, indicating convergence between network-driven and patient-centered strategies. Several of these genes were also identified by burden testing. Because the burden analysis was performed on NHC-selected genes, these results are not independent and should be considered suggestive. However, this concordance illustrates the potential value of integrating orthogonal analytical approaches when analyzing sparse genomic data.

The decision tree analysis also identified *DOCK10* as the primary discriminator between severe and moderate cases. The root split was driven by the burden of rare variants with CADD ≥ 15 or unannotated variants within this gene. *DOCK10* encodes a guanine nucleotide exchange factor involved in cytoskeletal organization and immune cell signaling. Although it did not emerge among the top genes in classical burden analyses, its central role in the decision tree suggests that it may capture nonlinear or combinatorial genetic effects not detectable through standard aggregation tests. Moreover, although gene-specific properties such as gene length, variant density, or annotation biases could in principle influence the number of variants observed within a gene, these properties are identical between the severe and the moderate groups, and therefore they are unlikely to account for the observed difference. This example highlights the complementary role of interpretable machine learning in uncovering latent genetic structures that extend beyond additive statistical models.

To assess the robustness of the decision-tree results, a leakage-controlled analysis was performed using a nested cross-validation design, in which all feature-construction and model-selection steps were repeated exclusively within training partitions. Under this conservative framework, out-of-sample discrimination between severe and moderate cases was not statistically supported.

These results indicate that the decision tree and the associated biclusters should not be regarded as validated predictive classifiers, but rather as a means of identifying candidate genes such as DOCK10 which is, in this case, those involved in cytoskeletal remodelling and immune cell signalling.

Overall, network-based aggregation, gene-level burden testing, and interpretable machine learning support a model in which rare variants influence COVID-19 severity through interconnected regulatory pathways governing transcription, immune response modulation, vesicular trafficking, and mitochondrial function. However, in vitro functional validation of key variants or statistical replication in larger-scale independent genomic cohorts are required to support our findings.

### Limitations

A key limitation in rare variant studies remains low statistical power owing to variant sparsity. Even with large sequencing efforts, most rare variants have modest effect sizes and require very large samples or aggregative tests to be robustly detected. Integrating the biological context through gene- or pathway-level aggregation has emerged as a proven strategy to enhance signal detection and interpretability in this setting, as shown across complex traits such as HDL levels, obesity, schizophrenia and cancer [54].

In addition, the sample size, although deeply characterized and enriched for phenotypic extremes, still limits the statistical power for rare variant detection; no a priori power calculation or minimum detectable effect estimate was performed, and, consistent with expectations for rare-variant burden analyses of this size, adequate power is likely only for large effect sizes. This may increase the number of false positives, particularly for variants or genes with low carrier frequencies. The same constraint affects the stability of the patient partition in the biclustering. Although the ten-cluster solution provided the most stable structure among those evaluated, biclustering results should be interpreted as exploratory. Therefore, replication in independent cohorts will be essential. Second, our analyses rely on in silico functional predictions and existing pathway and network annotations, which may incompletely capture regulatory and context-specific effects; functional studies will be needed to confirm the biological impact of the identified variants, genes and pathways. Third, like most rare variant association studies, our approach may miss contributions from ultra-rare or non-coding variants not well captured by current models and annotations and from gene–gene or gene–environment interactions that require substantially larger datasets to be interrogated. Finally, gene prioritisation via NHC and the subsequent association testing were performed on the same 191 participants, and the reported genomic inflation factor cannot fully exclude the resulting selection bias. While our directional label-inversion strategy discards clusters lacking specificity to the severe phenotype, it does not constitute an independent validation of the prioritised genes. The optimal validation strategy would be a discovery–validation split; however, given that NHC targets heterogeneous rare-variant signals, splitting our already limited cohort would reduce carrier counts per gene set and substantially lower power, so we consider this feasible only with larger sample sizes.

## 4. Methods and Materials

### 4.1. Dataset

The cohort analyzed in this study has previously been described in detail [24]. Briefly, 200 COVID-19 patients were recruited at the Azienda Unità Sanitaria Locale (AUSL)-IRCCS (Reggio Emilia, Italy) during the pandemic (March 2020–May 2020). Patients were classified as “severe” or “moderate” on the basis of previously published clinical criteria [55]. In particular, severe cases required hospitalization with noninvasive ventilation and/or intubation, whereas moderate cases were managed at home with symptomatic treatment only. The present work is based on the reanalysis of previously collected and anonymized data.

### 4.2. Whole-Genome Sequencing

Genomic DNA was extracted from peripheral blood via the QIAamp DNA Blood Mini Kit (QIAGEN, Venlo, The Netherlands) and quantified via both the NanoDrop system and the Qubit system following standard protocols. Whole-genome sequencing was then performed in a NovaSeq 6000 System (Illumina, Inc., San Diego, CA, USA) for each DNA sample. The quality of the sequencing data was evaluated via the FastQC tool [56].

The raw sequencing data were processed via the Illumina DRAGEN Germline Pipeline (v3.9.5) (Illumina, Inc., San Diego, CA, USA) [57], following GATK Best Practices [58], and aligned with the GRCh38 (hg38) reference genome to identify single nucleotide variants (SNVs) and small insertions/deletions (indels).

Joint variant calling was performed via Illumina DRAGEN Joint Genotyping (v3.9.5). Low-quality variants, defined according to standard thresholds [59], were excluded to minimize false positives. Autosomal variants deviating from the Hardy–Weinberg equilibrium in moderate cases (*p* < 10^−6^) were also removed as part of the quality control procedures, given that extreme departures from HWE are commonly associated with genotyping inaccuracies in rare variants.

Variant annotation was conducted with the Ensembl Variant Effect Predictor (VEP) v100.2 (EMBL-EBI, Hinxton, UK) [60], incorporating allele frequencies from gnomAD v3.0 [61], CADD pathogenicity scores v1.7 [62] and splice impact prediction from SpliceAI v1.3 [63]. Variants were further annotated for clinical significance via the ClinVar database [64]. For the analysis aimed at investigating potential germline mutations and/or a common dysregulated pathway, only rare variants—either absent from GnomAD or with a minor allele frequency (GnomADg_AF) < 0.01—restricted to coding regions and their ±100 bp flanking exonic sequences were considered.

To search for potentially causative or pro-inflammatory genetic variants of severe or moderate COVID-19, an in silico gene panel was set up that included genes previously reported in the literature and whose involvement in SARS-CoV-2 infection had already been suggested by the presence of susceptibility variants (Table 2).

### 4.3. Network-Based Heterogeneity Clustering

To investigate genetic factors associated with disease severity, the network-based heterogeneity clustering (NHC) algorithm v.2 [124] was applied as an exploratory, hypothesis-generating approach. NHC identifies groups of genes and individuals sharing potentially deleterious variants by creating clusters of individuals carrying functionally connected variants, an aspect not captured by classical burden tests.

Variants are first aggregated and mapped to their respective genes, after which biologically related genes are grouped across individuals within the background network. The analysis was conducted on the whole-genome sequencing data of 191 individuals (96 moderate and 95 severe cases, hereinafter referred to as controls and cases, respectively), with a focus on rare, high-quality variants. Genotypes were set to missing if they exhibited a low depth of coverage (DP < 7 for SNPs and DP < 10 for InDels), low genotype quality (GQ < 20), or unbalanced allele depth (AB < 0.15 or >0.85 for SNPs and AB < 0.20 or >0.80 for InDels). For Y and mitochondrial chromosomes, heterozygous genotypes were also set to missing. Variants that had >5% missing values after initial filtering were removed. Samples showing sex (chromosome X homozygosity > 0.2 for females or <0.8 for males), high relatedness (IBD > 0.185), divergent ancestry based on multidimensional scaling with the HapMap3 reference, abnormal heterozygosity (±3 SD from the mean), or genotype missingness > 3% were excluded. Following these standard quality control steps [59], the cohort was reduced from 200 to 191 individuals. Age, sex, and complications were previously assessed as potential covariates of COVID-19 severity in this cohort and found not to be significantly different between cases and controls [24]. Furthermore, the current implementation of NHC does not support the inclusion of clinical or demographic covariates; no covariate adjustment was therefore performed in the present analysis. In accordance with the NHC tool, the analysis was performed via both case–control and case–only (severe + moderate patients) models, starting from a connectivity gene network created from STRING [125], Reactome [126], BioGRID [127], and IntAct [128], and focusing on likely harmful, high-impact variants (CADD ≥ 15 or unannotated). The final output of the NHC comprises prioritised gene clusters derived from genes carrying variants in the case–cohort, with cluster-level *p* values ≤ 0.05 compared with those of the control cohort, and at least one pathway or gene ontology term enriched with *p* values ≤ 1 × 10^−5^. Because NHC does not intrinsically assign directionality to enrichment, both case–control contrasts (severe vs. moderate and the opposite) were tested, and only clusters significant in one direction and not in the reverse were retained to limit non-specific enrichment. As gene prioritisation and subsequent testing were performed on the same cohort, all resulting genes and pathways are treated as post-selection exploratory hypotheses.

### 4.4. Genetic Association Analysis

Rare variant association analysis was performed via RVTESTS [129], which focuses exclusively on genes identified through NHC clustering. Variants were preannotated via the ANNO pipeline [130] and analyzed with multiple association models, including kernel-based methods (SKAT, SKAT-O), burden tests (ExactCMC), and variable threshold tests (price model, with nPerm = 10^8^, alpha = 0.001, beta1 = 1, beta2 = 20). The latter was performed using the following RVTESTS command: “*rvtest --inVcf <vcf> --out <out> --pheno <pheno> --vt “price[nPerm=100,000,000:alpha=0.001:beta1=1:beta2=20]” --geneFile <geneFile> --gene <gene list>*”. For each gene, RVTESTS reports NumGreater and NumEqual as the number of permutations in which the permuted test statistic exceeded or equalled, respectively, the observed statistic, from which the permutation *p* value (Perm*p* value) is derived relative to the total number of permutations performed. *p* values were adjusted for multiple testing via the false discovery rate (FDR), and the genomic inflation factor (λ) was calculated to assess potential result inflation. Manhattan and Q–Q plots were generated via the qqman R package v.4.4.1 [131].

### 4.5. Biclustering Analysis

Using in-house software, the whole multisample vcf was transformed into a gene-level count matrix. Variants were summed by gene and stratified according to three criteria: (i) pathogenicity: variants were included if they had a CADD score ≥ 15 or not scored or were reported as pathogenic/likely pathogenic in ClinVar; (ii) zygosity: variants were distinguished as homozygous or heterozygous; and (iii) variant consequences according to Ensembl VEP classification: coding (frameshift, missense, protein-altering, synonymous), start/stop codon–affecting, splice-site, UTR (5′, 3′), NMD transcript, intronic, regulatory, noncoding transcript, and intergenic categories. The final gene-level count matrix consisted of one row per sample and, for each gene, multiple columns corresponding to all possible combinations of pathogenicity, zygosity, and variant consequences, each containing the number of variants observed for that feature. The total number of features per sample was 2,353,113.

Unsupervised biclustering analysis was employed to identify subsets of patients and variants exhibiting coherent patterns across the gene-level variant count matrix, referred to as biclusters, which differ from all other subsets. Biclustering encompasses computational techniques that iteratively detect biclusters by optimizing a relevance criterion that captures pattern coherence or similarity across both rows and columns of a data matrix simultaneously. These methods rely on heuristics that include greedy, divide-and-conquer, exhaustive enumeration, and distribution parameter identification [132]. The analysis used the SpectralBiclustering function from the scikit-learn package (v1.0.2) [133].

To assess whether the biclustering results depended on the prespecified number of clusters, we performed an outcome-independent stability analysis comparing solutions with n_clusters (K) = 5, K = 10, and K = 15. Spectral biclustering was performed according to the checkerboard model proposed by Kluger et al. [134], using the implementation available in the scikit-learn library [133]. In this implementation, an integer value K specifies K row clusters and K column clusters; it does not represent a maximum number of clusters. Clinical severity labels were not used to fit the biclustering models, assess their stability, or select the clustering resolution.

For each value of K, SpectralBiclustering was applied using bistochastic normalization and 10 k-means initializations (n_init = 10). Reproducibility with respect to stochastic initialization was evaluated by fitting the algorithm five times to the complete patient-by-variant matrix using different random seeds.

Agreement between every pair of solutions was quantified using the Adjusted Rand Index (ARI) [135], separately for the patient and variant partitions. The ARI measures agreement between two partitions while correcting for agreement expected by chance and is invariant to arbitrary permutations of cluster labels.

Robustness to changes in cohort composition was additionally evaluated using participant subsampling, following the general stability-based framework in which reliable clustering structures are expected to persist following controlled perturbations of the input data [136,137]. For each value of K, five subsampled datasets were generated by randomly retaining, without replacement, 80% of the participants. Spectral biclustering was independently refitted to each subsample.

For each subsampled solution, the patient-cluster labels were compared with those obtained from the corresponding full-data reference solution, considering only the retained participants. Because the complete set of genomic features was maintained in each subsample, variant-cluster labels were compared across all variants. Agreement was quantified using separate patient- and variant-partition ARIs.

For each candidate value of K, we calculated the mean initialization ARI for the patient and variant partitions and the mean and standard deviation of the corresponding subsampling ARIs. As a descriptive conservative summary, joint subsampling stability was defined as the lower of the mean patient- and variant-partition ARIs.

Because high stability may result from a nearly degenerate partition dominated by a single large cluster, the minimum, median, and maximum patient-cluster sizes were also examined. The clustering resolution was therefore evaluated by considering both stability and cluster-size balance, rather than by selecting the solution showing the strongest association with clinical severity.

The statistical associations between biclusters or decision tree stratification and disease severity were assessed via Fisher’s exact test with Monte Carlo simulation (10,000 replicates) using the Fisher test function from the stats R package.

A multivariable logistic regression model was fitted with disease severity as the dependent variable and bicluster membership, sex, age, and comorbidity as independent variables [138]. The overall adjusted contribution of bicluster membership was assessed using a likelihood-ratio test comparing the full model with an otherwise identical nested model excluding the bicluster term [139].

*p* values lower than 0.05 were considered statistically significant. A decision tree classifier using the Scikit-learn package (v1.0.2) was subsequently trained to stratify patients into biclusters enriched for severe or moderate phenotypes, with the aim of identifying patterns of patient stratification associated with disease severity. Hyperparameter tuning was performed via a grid search with fivefold cross-validation, using the following grid: max_depth: [2,3,4,5,6,7,8,9,10], min_samples_leaf: [5,10,15,20], and criterion: [“gini”, “entropy”]. Bar plots were generated with GraphPad Prism version 8.0.0 for Windows (GraphPad Software, Boston, MA, USA; www.graphpad.com).

### 4.6. Sensitivity Analysis of the Generalizability of the Bicluster-Derived Features

Because the decision tree was originally intended as an exploratory method for characterizing the variants contributing to patient stratification, it was not designed as a clinical prediction model. Nevertheless, to assess whether the bicluster-derived feature structure generalized to previously unseen participants, we performed a leakage-controlled nested cross-validation sensitivity analysis. To obtain an estimate of out-of-sample performance, we implemented nested stratified five-fold outer cross-validation and stratified four-fold inner cross-validation, in which the inner loop was used for model and hyperparameter selection and the outer loop was used exclusively for performance estimation [140,141].

Within each inner training partition, decision-tree hyperparameters were selected by maximizing the mean Matthews correlation coefficient. After hyperparameter selection, the complete feature-construction procedure was refitted using the corresponding outer-training partition, and the resulting model was evaluated once on the untouched outer-test partition. Out-of-fold predictions from the five outer folds were pooled to calculate the Matthews correlation coefficient, balanced accuracy, ROC-AUC, accuracy, sensitivity, specificity, and the confusion matrix. Ninety-five percent confidence intervals were estimated from 2000 patient-level non-parametric bootstrap resamples [141].

## 5. Conclusions

Our study was conducted on a targeted population; specifically, the Italian population. It aims to propose a possible approach for investigating the role of rare variants in complex diseases, such as COVID-19. In the case of a small sample group, whether due to the nature of the disease or to limitations (e.g., sequencing costs, data accessibility), the combined approach we propose could be a viable option.

The study design compares moderate cases with severe cases but omits asymptomatic or healthy controls, thus limiting the broader interpretation of baseline genetic susceptibility. The two groups, however, were selected not to identify genetic susceptibility variants, but rather those that modulate the phenotype. Therefore, despite their small size, the “moderate” and “severe” groups used are potentially the most suitable for exploring rare variants associated with phenotype modulation.

In particular, in this study, we investigated the contribution of rare genetic variants to COVID-19 severity in a small yet clinically and geographically homogeneous cohort of Italian patients. Two complementary analytical strategies were employed: first, pathway enrichment analysis followed by a gene-level association test on the prioritised genes; second, a machine learning approach integrating unsupervised biclustering with a decision tree classifier to uncover patterns of genetic contribution among clinical subgroups was used. As prioritisation and testing were performed on the same cohort, all reported genes and pathways should be regarded as post-selection exploratory hypotheses, requiring replication in independent cohorts. Taken together, our findings emphasize the importance of methodological integration and of interpretable computational approaches for generating testable hypotheses on the genetic architecture of multifactorial diseases, to be tested in the future, in larger-scale studies.

## Figures and Tables

**Table 1:** Statistically significant genes identified via the variable threshold method.

Gene	NumVar	NumPolyVar	OptFreq	Zmax	Stat	NumGreater	NumEqual	Perm*p* Value	fdr.*p*
EIF4G3	90	77	0	32.69	32.69	81	6	8.4 × 10^−7^	5.9 × 10^−4^
GTF2F2	26	24	1.04 × 10^−2^	30.79	30.79	705	5	7.1 × 10^−6^	2.5 × 10^−3^
LY96	5	3	2.62 × 10^−3^	28.02	28.02	3447	482	3.7 × 10^−5^	8.6 × 10^−3^
COX6A1	2	1	0	25.67	25.67	9251	6567	1.3 × 10^−4^	2.2 × 10^−2^
STK11	25	23	1.05 × 10^−2^	24.92	24.92	16,829	4819	1.9 × 10^−4^	2.7 × 10^−2^

**Table 2:** Gene panel. List of the genes (“Gene” column) included in the in silico genes panel. The genes are grouped by category (“Category” column) based on their function and are involved in SARS-CoV-2 infection, as suggested by the presence of previously reported susceptibility variants (“Reference” and “PMID ref” columns).

Category	Gene	Reference	PMID
Signalling and cellular metabolism associated with ACE2/TMPRSS2 axis	ACE	[65]	33117379
	ACE2	[66]	33092637
	ADAM17	[67]	33438249
	AGTR1	[68]	35120165
	TMPRSS2	[69]	32661206
		[70]	32501810
		[71]	33748697
		[72]	32664879
		[73]	32249956
		[74]	34668773
	CLTRN	[75]	33837377
	LZTFL1	[76]	32998156
	SLC6A20	[77]	32558485
	LOC107986083	[78]	33307546
	FOXP4-AS1	[79]	34237774
	CACFD1	[80]	37964352
	PLEKHA4		
	LOC105376940	[81]	33101356
Complement System	C2	[82]	34446527
	C3	[83]	41039248
	C4A	[84]	37376569
	C4B	[85]	36805858
	C5	[86]	35967527
	C6		
	C7		
	C8A		
	C8B		
	C1QA		
	C1QB		
	C1QC		
	C4BP		
	CFB		
	CFHR1	[87]	33643292
	CFHR3		
	CFHR2		
	CFHR4		
	CFHR5		
	CD55-DAF	[88]	35064759
	CFH		
	CFI		
	CD46		
Chemochine e Citochine	CCL2	[89]	35753602
		[90]	25818534
		[91]	26524966
	IL4		
	CCL5	[92]	35091748
	IL18	[93]	34911594
	IL1A	[94]	16652313
	IL10RB	[75]	33837377
	TNF	[95]	18312678
	IFNG	[96]	16672072
	IFNA10	[97]	35255492
	IFNL4	[98]	34707167
Toll-Like Receptors	TIRAP	[99]	34995294
	TLR2	[100]	36825132
		[91]	26524966
	TLR3	[27]	32972995
	TLR9	[101]	35538614
	TLR7	[102]	33650967
	TLR8-AS1	[66]	33092637
		[67]	33438249
Interferon-related	IFNAR1	[27]	32972995
	IFNAR2	[75]	33837377
	IRF3		
	IRF7		
	UNC93B1		
	IFITM3	[103]	30662816
		[104]	33396837
		[105]	34870250
		[106]	36423162
		[66]	33092637
	OAS1	[107]	35835913
	MX1	[108]	20462354
		[69]	32661206
		[109]	34581622
	OAS3	[78]	33307546
	DDR1	[110]	34071309
	TLL1		
	ACSL6	[97]	35255492
	LOC107986082		
	PLSCR1		
	THBS3		
	ISG15	[111]	35764457
	ILRUN	[80]	37964352
Glutathione transferase omega	GSTO1	[112]	35330457
	GSTO2		
RNA sensor MDA5	IFIH1	[113]	35060899
Metalloproteinase	ADAMTS13	[88]	35064759
Mannose-binding lectin	MBL	[90]	25818534
		[114]	34628288
		[115]	33745077
	MBL2	[116]	35102342
	LOC105378308		
Vitamin D binding protein	DBP	[117]	32770768
Dipeptidylpeptidase	DPP4	[118]	33774023
		[69]	32661206
	DPP9	[78]	33307546
		[79]	34237774
Human Leukocyte Antigen	HLA-DRB1	[119]	33896121
	HLA-A	[120]	33298875
	HLA-G	[78]	33307546
	CCHCR1		
Serum glycoprotein	AHSG	[121]	33933633
Apolipoprotein	APOE	[122]	32451547
		[66]	33092637
Immune Receptors	CCR1	[75]	33837377
	CCR9	[77]	32558485
	CXCR6		
	XCR1		
	FCGR2	[123]	35149195
Oxidative Stress and Inflammation	KEAP1	[75]	33837377
	PDE4A		
	LOC105374493		
	LOC105377067		
Endothelial regulation/inflammation and coagulation control	THBD	[88]	35064759
	NOTCH4	[78]	33307546
	TMEM189-UBE2V1	[120]	33298875
		[66]	33092637
Mucosal barrier/Epithelial innate defense	MUC5B	[22]	35771531
Autophagy	FYCO1	[77]	32558485
Various Enzymes	ABO	[77]	32558485
		[80]	37964352
		[66]	33092637
	TYK2	[78]	33307546
	FDX2	[79]	34237774
	KANSL1		

## Data Availability

The aggregated dataset generated and analyzed during the current study has been deposited in the European Variation Archive (EVA), with accession number PRJEB115165.

## Supplementary Materials

The following supporting information can be downloaded at: https://www.mdpi.com/article/10.3390/ijms27209152/s1.
